# Carbon dynamics of a controlled peatland rewetting experiment in the Norwegian boreal zone

**DOI:** 10.1038/s41598-025-30836-2

**Published:** 2025-12-17

**Authors:** Michael A. H. Bekken, Astrid Vatne, Poul Larsen, Andreas Ibrom, Klaus Steenberg Larsen, Bo Elberling, Kristoffer Aalstad, Sebastian Westermann, Jacqueline K. Knutson, Lena M. Tallaksen, Peter Dörsch, Peter Horvath, Anders Bryn, Norbert Pirk

**Affiliations:** 1 Department of Geosciences, University of Oslo, Oslo, Norway; 2 Centre for Biogeochemistry in the Anthropocene, University of Oslo, Oslo, Norway; 3 DMR, Nørresundby, Denmark; 4 Department of Environmental and Resource Engineering, Danish Technical University, Lyngby, Denmark; 5 Department of Geosciences and Natural Resource Management, University of Copenhagen, Copenhagen, Denmark; 6 Faculty of Environmental Sciences and Natural Resource Management, Norwegian University of Life Sciences, Ås, Norway; 7 Natural History Museum, University of Oslo, Oslo, Norway

**Keywords:** Peatlands, Rewetting, Eddy covariance, Greenhouse gas fluxes, Terrestrial carbon cycling, Norway, Biogeochemistry, Climate sciences, Ecology, Ecology, Environmental sciences

## Abstract

A controlled peatland rewetting experiment was conducted on two adjacent drained peatland sites in southeastern Norway. Eddy covariance monitoring of CO_2_ and CH_4_ fluxes at both sites began in 2019. In 2021, the Treatment Site was rewetted while the Control Site remained drained. Using nine environmental variables and the processed flux data as training data, Bayesian Additive Regression Tree (BART) models were used to generate annual flux balances for CO_2_ and CH_4_. The 4-year mean annual flux at the Control Site was 17.3 ± 10 g CO_2_-C m^− 2^ yr^− 1^ and 4.6 ± 0.1 g CH_4_-C m^− 2^ yr^− 1^. At the Treatment Site, the 2-year mean annual flux before the rewetting was 12.2 **±** 3.8 g CO_2_-C m^− 2^ yr^− 1^ and 1.8 **±** 0.04 g CH_4_-C m^− 2^ yr^− 1^. In the first year after rewetting the annual flux was 53.3 **±** 13 g CO_2_-C m^− 2^ yr^− 1^ and 3.8 **±** 0.3 g CH_4_-C m^− 2^ yr^− 1^, and in the second year after rewetting the annual flux was 41.2 **±** 18 g CO_2_-C m^− 2^ yr^− 1^ and 3.4 **±** 0.4 g CH_4_-C m^− 2^ yr^− 1^. BART counterfactual modeling was able to estimate the effect of the rewetting on CO_2_ and CH_4_ fluxes. Two years after the rewetting, the BART counterfactual modeling estimated that the cumulative fluxes had increased by 80.3 ± 49 g CO_2_-C m^− 2^ and 3.4 ± 0.47 g CH_4_-C m^− 2^ because of the rewetting. Carbon flux monitoring of both sites is ongoing as the Control Site remains drained and the soil and vegetation at the Treatment Site continues to adjust to the altered hydrological regime after rewetting.

**Supplementary Information:**

The online version contains supplementary material available at 10.1038/s41598-025-30836-2.

## Introduction

Starting approximately 12 000 years ago, present day northern peatlands began to form and accumulate large quantities of carbon, significantly impacting the global C budget^1^. The average carbon flux in northern peatlands across the Holocene is estimated to be -18.6 g C m^− 2^ yr^− 1^, with the fastest rates of carbon accumulation occurring in the early Holocene (-25 g C m^− 2^ yr^− 1^)^1^. Today, estimates of the carbon stock of northern peatlands are as high as 1055 Gt C^2^. However, draining of both northern and tropical peatlands for agriculture and forestry combined with the extraction of peat as a fuel source has likely turned peatlands from a global carbon sink to a carbon source^3^. Draining of peatlands increases carbon emissions through increasing the rate of oxidative microbial decomposition, increasing dissolved carbon loss, and making peatlands more vulnerable to fire^4^. At present, drained peatlands emit approximately 2 Gt CO_2_ annually, which accounts for 5% of all anthropogenic greenhouse gas emissions^5^.

Norway has the third largest extent of peatlands in Europe, after Finland and Sweden. However, given Norway’s topographical complexity and ecosystem diversity, estimating peatland area in the country is challenging. The Norwegian area frame survey of land cover estimated that Norwegian peatlands cover 41 655 km^2^^6^, while Moen et al.^7^ estimated the area of peatland in Norway to be 44 700 km^2^. Estimates of the drained peatland area in Norway also vary. Kløve et al.^8^ estimated that 5050–5700 km^2^ of peatlands have been drained in Norway: 4200 km^2^ for forestry and between 850 and 1500 km^2^ for agricultural purposes. Synthesizing the work of Johansen^9^, Johansen^10^, and Moen and Øien^11^, Moen et al.^7^ estimated that the area of drained peatland in Norway to be 7000 km^2^. These estimates primarily define peatlands as lands where peat reaches at least 30 cm deep. Conversely, the 2023 National Inventory Report (NIR) for Norway, which reports the land sector carbon balance of Norway to the United Nations Framework Convention on Climate Change (UNFCCC), and which defines forested organic soils as those reaching 40 cm deep, estimated a drained organic soil area of 2317 km^2^^12^. However, if drained forested mineral soils are included in this estimate, the total area of drained soil becomes 7790 km^2^, an estimate much closer to Moen et al.^7^.

Previous research suggests that drained peatlands could contribute significantly to Norway’s annual carbon emissions. Using tier-1 IPCC emission coefficients, the 2023 NIR for Norway estimated that drained organic soils in Norway emit 4.65 million tons of CO_2_e yr^− 1^, or 9% of Norway’s annual carbon emissions, which were 49.2 million tons CO_2_e yr^− 1^ in 2021^12^. Joosten et al.^13^ estimated that 3618 km^2^ of peatlands are drained in Norway and that these lands emit 5.55 million tons CO_2_e yr^− 1^. Regardless, both estimates by the NIR^12^ and Joosten et al.^13^ are made with tier-1 IPCC coefficients, which do not incorporate country-specific data and thus are inherently uncertain. Grønlund et al.^14^ estimated carbon emissions from peat soils used for agricultural purposes in Norway via long-term monitoring of subsidence rates, mineral content, and CO_2_ flux measurements. The authors estimated the area of agricultural peat soil to be 630 km^2^ and the total C loss to be approximately 1.8 to 2 million tons CO_2_e yr^− 1^ (600 g C m^− 2^ yr^− 1^). Higher resolution estimates of carbon emissions from all drained Norwegian peatlands, not only agricultural peatlands, are currently not available in part because only very few studies have measured carbon fluxes in these landscapes in Norway^8^.

Given the potential for drained peatlands to be a significant contributor to Norway’s carbon emissions, the Norwegian Environment Agency has prioritized peatland rewetting work to reduce greenhouse gas emissions and improve ecosystem health. As of 2020, 80 sites have been restored, and more sites are to be restored in the coming decades^15^.

While results of peatland rewetting vary, previous research indicates rewetting most often leads to a reduction in vertical carbon fluxes of CO_2_^16,17^. However, CH_4_ fluxes commonly increase after rewetting, though emission levels are highly dependent on vegetation composition and level of ecological succession^18^. Methane emissions after rewetting can also lag because of long term suppression of methane producing microorganisms^19^. As such, it is important that long term studies (i.e., longer than three years) are conducted to better understand the carbon dynamics of restored peatlands and their true potential for climate mitigation. However, long-term monitoring programs for restored peatlands are rare, which is a limitation of current research^16^.

Waterborne lateral carbon fluxes in peatlands can be equal to or greater than vertical carbon fluxes and are important to consider when establishing a carbon balance of peatland ecosystems^20,21^. After peatland rewetting, dissolved organic carbon (DOC) fluxes can be particularly high if a high volume of soil is left exposed^22^. However, lateral fluxes in peatland ecosystems have been studied less than vertical carbon fluxes^23^. Lateral carbon transport includes DOC, dissolved inorganic carbon (DIC), particulate organic carbon (POC), dissolved CO_2_ gas and dissolved CH_4_ gas. DOC is commonly the largest source of carbon transport from peatlands. Once transported offsite, on average between 80 and 100% of DOC is eventually respired and emitted to the atmosphere as CO_2_^22^.

This research is motivated by current estimates which indicate carbon emissions from drained peatlands in Norway are a major component of the country’s land sector carbon budget^13^. However, the current lack of field-based research monitoring such emissions in Norway limits the accuracy of these estimates. The timely aim of this project is to investigate to what extent peatland rewetting in Norway would have its intended effect of carbon emission reduction as envisaged by Husby^15^.

To achieve these aims a controlled peatland rewetting experiment is being conducted, in which two adjacent peatland sites–the Treatment Site and Control Site–were selected and monitored for vertical CO_2_ and CH_4_ fluxes using eddy covariance. After two years of monitoring, the Treatment Site was rewetted while the Control Site remained drained. This paper focuses on the short-term response in vertical CO_2_ and CH_4_ fluxes at the Control Site and Treatment Site two years before and two years after the rewetting. Additionally, vegetation mapping, water table depth monitoring, soil analyses, and coarse estimates of DOC fluxes were conducted at both sites. Monitoring at both sites is ongoing to develop a longer time series of flux data that will be used to study the longer-term effects of rewetting.

## Methods

### Site description, drainage conditions, experimental design, and timeline

**Fig. 1:**
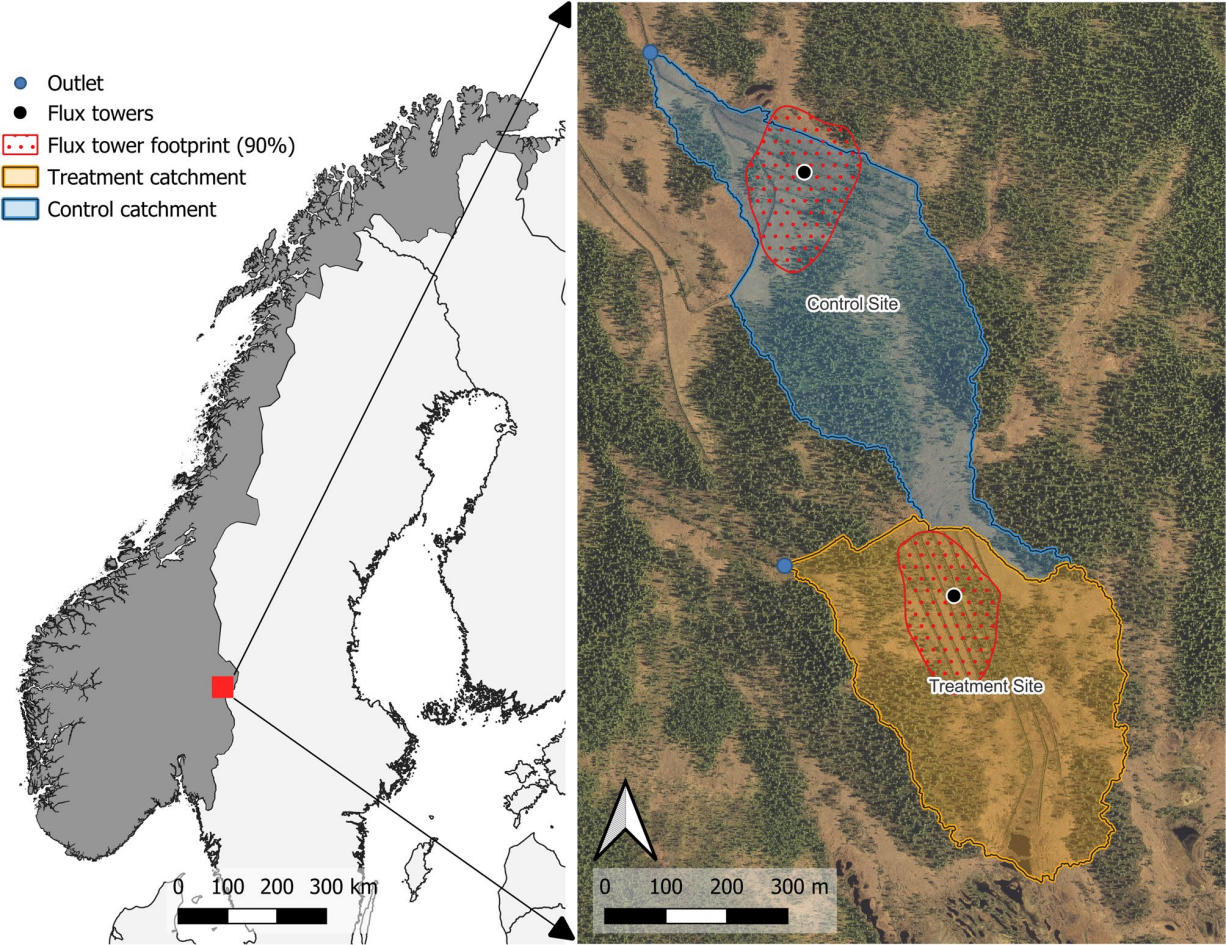
The Regnåsen and Hisåsen Nature Reserve is in southeastern Norway. Catchments of the Control Site and Treatment Site are shaded in blue and orange, respectively. The outlet of each site’s catchment is marked with a blue dot. A black dot marks the placement of the two flux towers. The 90% footprint of the two flux towers is delineated by the red dotted polygon. The map was created in QGIS 3.30 (QGIS.org, 2023).

To date, most peatland rewetting projects by the Norwegian Environment Agency have been conducted in southeastern Norway. Consequently, drained peatland sites in this region were prioritized. The selected sites needed to be relatively close to share a single off-grid power supply and be easily accessible for routine maintenance of the measurement equipment and power systems. Two peatland sites in the Regnåsen and Hisåsen Nature Reserve (Trysil Municipality, Innlandet County) met all selection criteria (Fig. 1).

Both the Control Site and Treatment Site were presumably drained for forestry. Aerial photography indicates that both sites were drained between 1956 and 1975^24^, though the exact year is uncertain due to a lack of historical records. The Control Site was drained by six parallel drainage ditches, spaced by 20–30 m (measured from the center of one drainage ditch to the next), and that range from approximately 1 to 1.5 m deep. The Treatment Site was drained with seven parallel drainage ditches, spaced by 15 to 25 m and that range from approximately 1 to 1.2 m deep.

An eddy covariance tower was installed at the center of both the Treatment Site (61.105185°N, 12.254734°E) and the Control Site (61.111581°N, 12.250595°E) in June 2019. The flux tower at the Treatment Site is located at 680 m asl. and the flux tower at the Control Site is located at 640 m asl. The drainage ditches at the Treatment Site cover 4.7 ha and 3.3 ha at the Control Site. The Treatment Site underwent rewetting from September to November 2021, while the Control Site remained drained. For consistency in tracking annual fluxes, 1 October 2021 is used as the rewetting date. Annual fluxes are reported for the two years before the rewetting (1 October 2019–30 September 2021), which is referred to as the Pre-Treatment Period. Similarly, annual fluxes are reported for two years after the rewetting (1 October 2021–30 September 2023), which is referred to as the Treatment Period. 2019/20 is used to represent the first year of the study from 1 October 2019–30 September 2020. The same notation is used to refer to the second (2020/21), third (2021/22), and fourth year (2022/23) of the study. However, there is one exception to this convention. Dissolved organic carbon (DOC) lateral fluxes were estimated on a standard calendar basis in 2021 and 2022.

### Regional geography, geology, climate, and ecosystem description

The Regnåsen and Hisåsen Nature Reserve is 15.7 km^2^ and ranges from 400 to 720 m asl. in elevation. The reserve was created in 2017 to preserve biological diversity, including the lichens, coniferous forests, and peatland types in the area^25^. The reserve is dominated by evergreen conifers with scattered peatlands appearing in flat or depressed landscapes. The geology is dominated by Precambrian rocks of the Baltic shield, predominantly slowly weathering quartzite and feldspar-dominated granites^26^. The Köppen-Grieger climate classification is subarctic (Dfc - cold, no dry season, cool summers)^27^. For the period between 1991 and 2020 the mean annual temperature was 3.4 °C and the mean annual precipitation was 946 mm^28^.

### Rewetting description

**Fig. 2:**
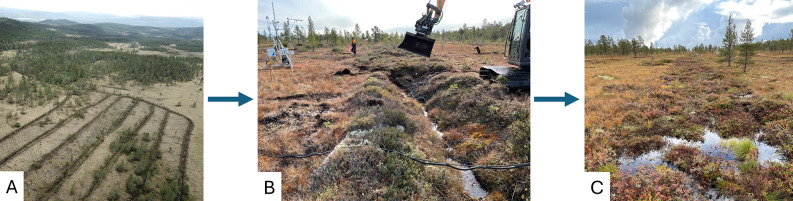
(**A**) The Treatment Site before rewetting, (**B**) rewetting Treatment Site in October 2021, and (**C**) and the completed rewetting in September 2022. Photo Credit: Norbert Pirk, Poul Larsen, and Michael Bekken.

From September to November 2021, the Treatment Site was rewetted though ditch blocking by the Norwegian Environment Agency (Fig. 2). Three hundred and sixteen peat dams at 40 cm elevation intervals were built using peat excavated from between the ditches. In total, 3 823 m of ditches were blocked that had previously drained the site. To minimize soil and vegetation disturbance the first step in the soil excavation process was to remove the grasses and shrubs, including their rootzones, and set them aside. The ditches were then filled. Once this was completed, the vegetation was replaced to its original location. Some of the trees growing on the site, primarily those on the edges of the ditches, were cut down and also used to fill the ditches. To our knowledge, the rewetting of the Treatment Site did not affect the Control Site.

### Peat coring

Between 6 and 8 August 2024, peat samples were collected at 5 cm intervals in three replicate profiles within a 10 × 10 m area at both the Treatment Site and Control Site. Coring was conducted manually in approximately 0.5 m increments. Cores were immediately photographed and subsequently cut in 5 cm intervals and transferred to plastic bags. Samples were kept cold at approximately 5 °C until they were processed in the laboratory.

Based on the total weight of the volume-specific samples before and after drying (48 h), the water contents and bulk densities were calculated. Soil pH was measured directly in soil water extractions, using a soil to water ratio of 1:5. Deionized water was added and the mix shaken for 1 h at room temperature, centrifuged and finally filtered through a 5 μm membrane filter before the pH measurement (pHenomenal pH 1100 LTM, VWR, Darmstadt, Germany). After drying the subsamples were finely ground and analyzed for carbon (C) and nitrogen (N) concentration using a Eurovector elemental analyzer (Redavalle, Italy) coupled to Isoprime (Isoprime Ltd., Cheadle Hulme, UK) isotope ratio mass spectrometer (IRMS). Depth-integrated soil C pools were calculated based on soil bulk density and content of soil C reported in four depths: 0–30, 30–50, 50–100 and 0–100 cm.

### Vegetation mapping

Vegetation at the site was mapped in-situ using field-computers, and polygon delineation assisted by aerial photos in QGIS. The vegetation was mapped in accordance with the *Nature in Norway* v.2.3 classification system^29^, which includes standardized mapping guidelines. Mapping was conducted in August 2022 over 0.25 km^2^ area surrounding each flux tower. The mapping scale was set to 1:20 000 with a minimum polygon size of 1 000 m^2^.

### Meteorological measurements and water table depth monitoring

Radiation was measured with a CNR4 radiometer (Kipp & Zonen, Netherlands). Air temperature and relative humidity were measured with a HMP155 (Vaisala, Finland). Soil temperature was measured using a Stevens HydraProbe sensor (Stevens Water Monitoring Systems Inc, USA). Five WatsonC loggers equipped with RKL-01 water table depth sensors (Hunan Rika Electronic Tech Co. Ltd) were installed in five water table depth monitoring wells at both the Treatment Site and the Control Site. For the location of the water table depth monitoring wells see Supplementary Information (Sect. 1, Table S1).

Gaps in the time series of the meteorological variables measured onsite (shortwave and longwave radiation, air temperature and pressure, vapor pressure deficit, wind speed) were filled using the bias-corrected ERA5-Land dataset^30^, downloaded from the Climate Data Store (CDS) of the Copernicus Climate Change Service (C3S). The ERA5-Land variables were first bias corrected by using a simple linear regression with the corresponding onsite variable. A random forest regression was then trained for each meteorological variable, using the bias-corrected ERA5-Land meteorological variables, time since rain, and growing degree days as predictors. Gaps in the onsite meteorological and soil temperature time series were then filled using the random forest regression^31^. Annual ET estimates were obtained by gap filling eddy covariance ET data with a random forest model using the gap-filled meteorological and surface variables as predictors.

### Eddy covariance measurements

To monitor vertical carbon fluxes of CO_2_ and CH_4_, eddy covariance flux towers were installed at each site on 19 June 2019. The flux towers are equipped with a HS50 sonic anemometer (Gill Instruments Ltd, UK), a Li-7200 infrared gas analyzer for CO_2_ and H_2_O mixing ratios, and a Li-7700 open-path gas analyzer for CH_4_ densities (LI-COR Biosciences, USA). These instruments were installed at a height of 2.8 m above ground level and sampled at 20 Hz. These raw data are processed in 30-minute intervals following the conventional eddy covariance method^32^ implemented in EddyPro version 6.2.0 (LI-COR Biosciences, USA). A block average Reynolds decomposition was used to extract turbulent fluctuations, and corrections were applied for anemometer tilt, a constant time lag compensation, and a high and low-pass filter correction^33,34^, as well as the Webb, Pearman, and Leuning (WPL) correction for CH_4_ fluxes^35^.

For quality control, the statistical tests proposed by Vickers and Mahrt^36^ were used on the raw data and the flagging system proposed by Foken and Wichura^37^ were used to filter out flux estimates affected by instrument errors (e.g., rain or frost on the anemometer) or unfavorable micrometeorological conditions (e.g., lack of stationarity or turbulent mixing at low wind speeds). Following Vickers and Mahrt^36^, the number of spikes and drop-outs, as well as the absolute limits, amplitude resolution, skewness and kurtosis, and discontinuities in the raw data time series were used for EC flux filtering. Data exceeding the threshold proposed in Vickers and Mahrt^36^ were discarded. In a manner consistent with Pirk et al.^38,39^ data was also discarded when the mean horizontal wind speed was below 1.5 m s^− 1^. All fluxes with quality flags 1 and 2 in the scheme by Foken and Wichura^37^ were also discarded.

Lastly, all data reported here uses positive numbers to represent carbon fluxes from the surface to atmosphere and negative numbers are used to represent carbon fluxes from the atmosphere to surface.

### Statistical flux modeling

All valid eddy covariance measurements were used as training data for a set of Bayesian Additive Regression Tree (BART) models. These BART models were introduced in the statistics literature by Chipman et al.^40^ and more recently reviewed by Hill et al.^41^. BARTs can be thought of as Bayesian (i.e., probabilistic) extensions of popular ensemble-based decision tree machine learning methods. The latter include random forests and extreme gradient boosting (XGBoost), both of which have shown promise for gap-filling eddy covariance data^38,42^. In this study, BARTs were chosen over a random forest or XGBoost approach because of their superior computational performance^43^, and because BARTs provide uncertainty quantification. Specifically, BARTs are sum-of-tree models more closely related to boosting than to bagging-based methods like random forests^40^. What sets BARTs apart from the aforementioned conventional ensemble-based decision tree methods is their foundation in Bayesian inference^44,45^ rather than traditional optimization techniques. Instead of producing a single forest based on an ensemble of decision trees, a BART model generates a posterior meta-ensemble of forests. This uncertainty-aware framework enhances robustness in decision-making and causal inference^41^.

In this study, BARTs were constructed in the probabilistic programming language PyMC^46^ as presented by Quiroga et al.^47^. Specifically, the BART models were trained to predict fluxes based on nine environmental variables. The environment variables used included shortwave and longwave incoming radiation, air temperature and pressure, vapor pressure deficit, wind speed, growing degree days, time since rain, and soil temperatures at five depths (5–70 cm). For further information about the BART model setup, see the Supplementary Information (Sect. 2).

For consistency between modeling of actual fluxes and counterfactual fluxes, the BART flux estimates, which matched the eddy covariance flux measurements closely, were also used even when valid measurements from the eddy covariance system existed. Summary statistics in the form of the mean, 5th percentile, and 95th percentile of the 200-member posterior ensemble of the BART predictions are used as the best estimate of actual fluxes.

### Counterfactual flux modeling

In the original experimental design, the continuously drained Control Site was intended to serve as the counterfactual to the rewetted Treatment Site. However, differences between the CO_2_ and CH_4_ fluxes between the two sites before the rewetting of the Treatment Site, meant that it was not possible to use the Control Site as a counterfactual representation of fluxes at the Treatment Site after rewetting. Instead, counterfactual BART modeling was used to develop best estimates of the effect of the rewetting on CO_2_ and CH_4_ fluxes.

A BART model was built for both the Treatment Site and the Control Site from the eddy covariance data from 1 October 2019 until the rewetting in October 2021; these are referred to as the Pre-Treatment Models. Separate BART models were built for both sites from eddy covariance data from 1 October 2021–1 October 2023; these are referred to as the Treatment Models.

To determine if the Pre-Treatment Models could accurately simulate fluxes during the Treatment Period at the Control Site, and to obtain a counterfactual estimate of what the fluxes would have been at the Treatment Site had the rewetting not occurred, we used the Pre-Treatment Models to simulate Treatment Period fluxes at both sites. To run this simulation, the Pre-Treatment Models were forced with nine environmental variables from the Treatment Period to make flux predictions. At the Control Site, we hypothesized that the Pre-Treatment Model simulation of the Treatment Period would match our best estimate of the actual Treatment Period fluxes derived from the Treatment Model. At the Treatment Site, we hypothesized that the Pre-Treatment Model simulation of Treatment Period would diverge from the actual fluxes as estimated by the Treatment Model, and that this divergence would be representative of the effect of the rewetting.

### Lateral carbon flux estimation methods

To estimate dissolved organic carbon (DOC) concentrations in the streams draining the Control Site and the Treatment Site, water samples were collected in 0.5 L bottles on seven different occasions at the Control Outlet and the Treatment Outlet (Fig. 1). Four sampling trips were made to take DOC samples before the rewetting in 2021 (22 June, 26 July, 28 July, and 30 July), and three trips were made to take samples after the rewetting in 2022 (9 September, 13 September, and 10 October). For further details about the water sampling, see Supplementary Information (Sect. 3).

A coarse estimate of DOC fluxes was made using Eq. 1.

1 $$\:{DOC}_{f}\:=\left(\frac{Q*{DOC}_{c}}{A}\right)$$

Where *DOC*_*f*_ is the lateral DOC flux (g C m^− 2^ yr^− 1^), where *Q* is the lateral discharge rate (m^3^ yr^− 1^), where *DOC*_*c*_ is DOC concentration (g C m^− 3^), and where *A* is catchment area (m^2^). Discharge was estimated using a simple water balance model. For further details about the method of discharge estimation, see Supplementary Information (Sect. 4).

## Results

### Vegetation mapping

The peatlands at Regnåsen and Hisåsen are formed by species-poor fens (V1-E-1) and swamp forests with a scattered tree-layer dominated by *Pinus sylvestris* (V2-E-1) and *Betula pubescens* (Table 1). These areas receive an influx of groundwater, but the slow weathering and nutrient poor bedrock provides low levels of basic plant nutrients. The peatlands of both ecosystem types are dominated by the vascular plants: *Trichophorum cespitosum*, *Eriophorum vaginatum*, *Carex puciflora*, *Andromeda polifolia* and *Vaccinium* species. The bottom-layer is dominated by *Sphagnum* species such as *S. compactum*, *S. tenellum*, *S. lindbergii* and *S. papillosum*^48^. On firm dry ground *Pinus sylvestris* form heather forests with *Pleurozium schreberi* (T4-E-4), whereas *Hylocomium splendens*, *Polytrichum* sp. and *Sphagnum* mosses form the bottom-layer on more moist ground (T4-E-1). The very small lakes (L) are typically dystrophic (high levels of dissolved humus and low oxygen levels), with species such as *Scheuchzeria palustris* along the water’s edge.

**Fig. 3:**
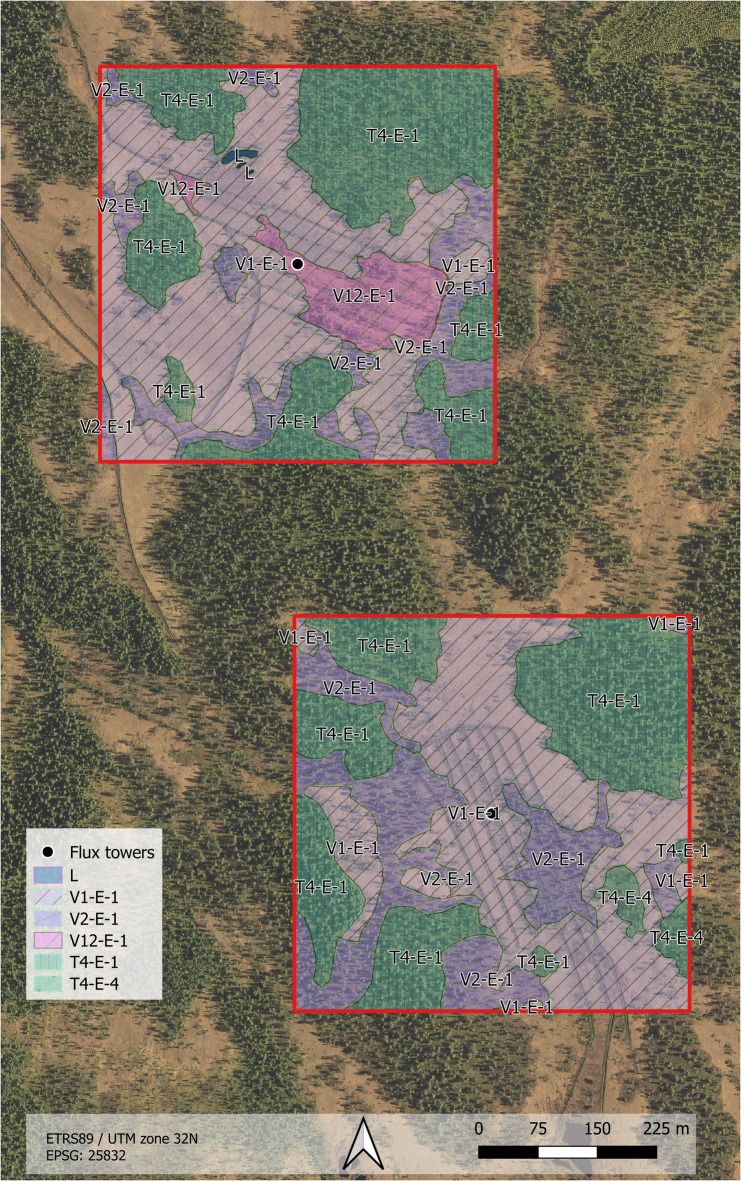
Nature in Norway (NiN) vegetation map of the Treatment Site (south) and Control Site (north) at a 1:20 000 scale. Vegetation mapping was conducted in August 2022, approximately 10 months after the rewetting of the Treatment Site. The map was created in QGIS 3.30^49^.

The mapping area at the Control Site consisted of predominantly species-poor fen systems (V1-E-1: 42%), blueberry forest (T4-E-1: 36%) and swamp forest (V2-E-1: 14) (Fig. 3). The Treatment Site consisted of predominantly species-poor fens (V1-E-1: 40%), blueberry forest (T4-E-1: 35%), and swamp forest (V2-E-1: 23%).

**Table 1:** Vegetation types of the treatment site and control site within the flux tower footprint (Fig. 1) and the mapping area (Fig. 3) as mapped in August 2022, approximately 10 months after the rewetting of the treatment site.

Common name	NiN mapping code unit	Treatment Site				Control Site			
		Mapping area		Flux Tower Footprint		Mapping Area		Flux Tower Footprint	
		Area (m^2^)	Percent (%)	Area (m^2^)	Percent (%)	Area (m^2^)	Percent (%)	Area (m^2^)	Percent (%)
Freshwater bottom systems	L	-	-	-	-	648	-	-	-
Heather forest	T4-E-4	6 029	2	-	-	-	-	-	-
Blueberry forest	T4-E-1	87 511	35	858	3	89 187	36	1 731	5
Species-poor fens	V1-E-1	99 087	40	22 993	73	103 988	42	24 086	69
Swamp forest	V2-E-1	57 367	23	7 335	24	35 802	14	2 178	6
Drained species-poor fens	V12-E-1	-	-	-	-	20 372	8	7 209	20
Total area		249 994	100	31 186	100	249 996	100	35 204	100

### The effect of the rewetting on the water table depth

**Fig. 4:**
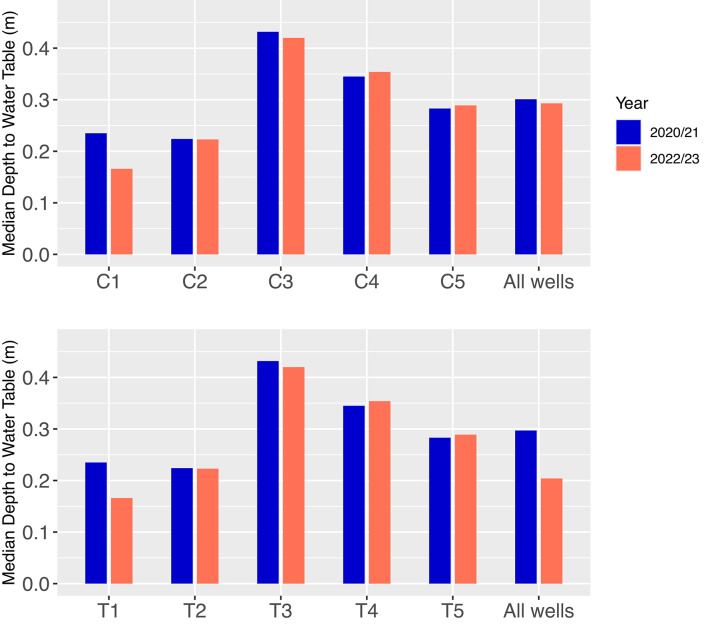
Median water table depth in five groundwater wells at the Control Site (C1 - C5) and at the Treatment Site (T1 - T5) and the median water table depth across all wells at each site.

Figure 4 compares the median water table depth in five wells at the Control Site and the Treatment Site in the year before the rewetting in 2020/21, and two years after the rewetting, in 2022/23. The median water table depth across all wells at the Control Site was 30 cm in the year before the rewetting and 29 cm two years after the rewetting. The median water table depth across all wells at the Treatment Site was 30 cm in the year before the rewetting and 21 cm two years after the rewetting. Therefore, as estimated by the five wells at the Treatment Site, the rewetting raised the median water table level by 9 cm.

### Soil characteristics

The peat characteristics, as measured after the rewetting of the Treatment Site, were similar at the two sites in pH and carbon content with both properties increasing slightly with depth (Table 2). However, the total carbon stock to 1 m depth was markedly higher at the Treatment Site than at the Control Site, particularly at depths below the depth influenced by rewetting (0.5–1 m). Overall, the peat can be characterized as an acidic, organic rich peat.

**Table 2:** Mean soil characteristics of three soil cores from the control site and three cores from the treatment site. Each core was analyzed in 5 cm depth increments but is reported here in four depth intervals (0–30, 30–50, 50–100, and 0–100 cm).

Depth (cm)	pH	Total C (%)	C/*N*	Bulk density (g cm^− 3^)	C stock (kg m^− 2^)
Control Site					
0–30	4.1 ± 0.2	48.1 ± 3.2	29.0 ± 14.1	0.06 ± 0.02	9.1 ± 0.9
30–50	4.2 ± 0.1	50.5 ± 1.5	30.1 ± 1.9	0.06 ± 0.01	6.1 ± 0.5
50–100	4.4 ± 0.1	50.6 ± 2.6	31.1 ± 3.0	0.07 ± 0.02	18.9 ± 0.4
0-100	4.3 ± 0.2	49.8 ± 1.8	30.3 ± 5.9	0.07 ± 0.02	34.1 ± 2.1
Treatment Site					
0–30	4.2 ± 0.1	49.4 ± 4.5	27.6 ± 9.0	0.08 ± 0.03	11.7 ± 0.8
30–50	4.3 ± 0.1	53.7 ± 3.3	31.5 ± 6.0	0.06 ± 0.01	6.9 ± 0.4
50–100	4.4 ± 0.2	54.9 ± 3.4	36.8 ± 11.4	0.10 ± 0.03	26.6 ± 0.4
0-100	4.3 ± 0.2	53.0 ± 3.9	33.0 ± 6.8	0.09 ± 0.03	45.2 ± 3.1

### Vertical CO_2_ fluxes

#### Instantaneous CO_2_ fluxes

**Fig. 5:**
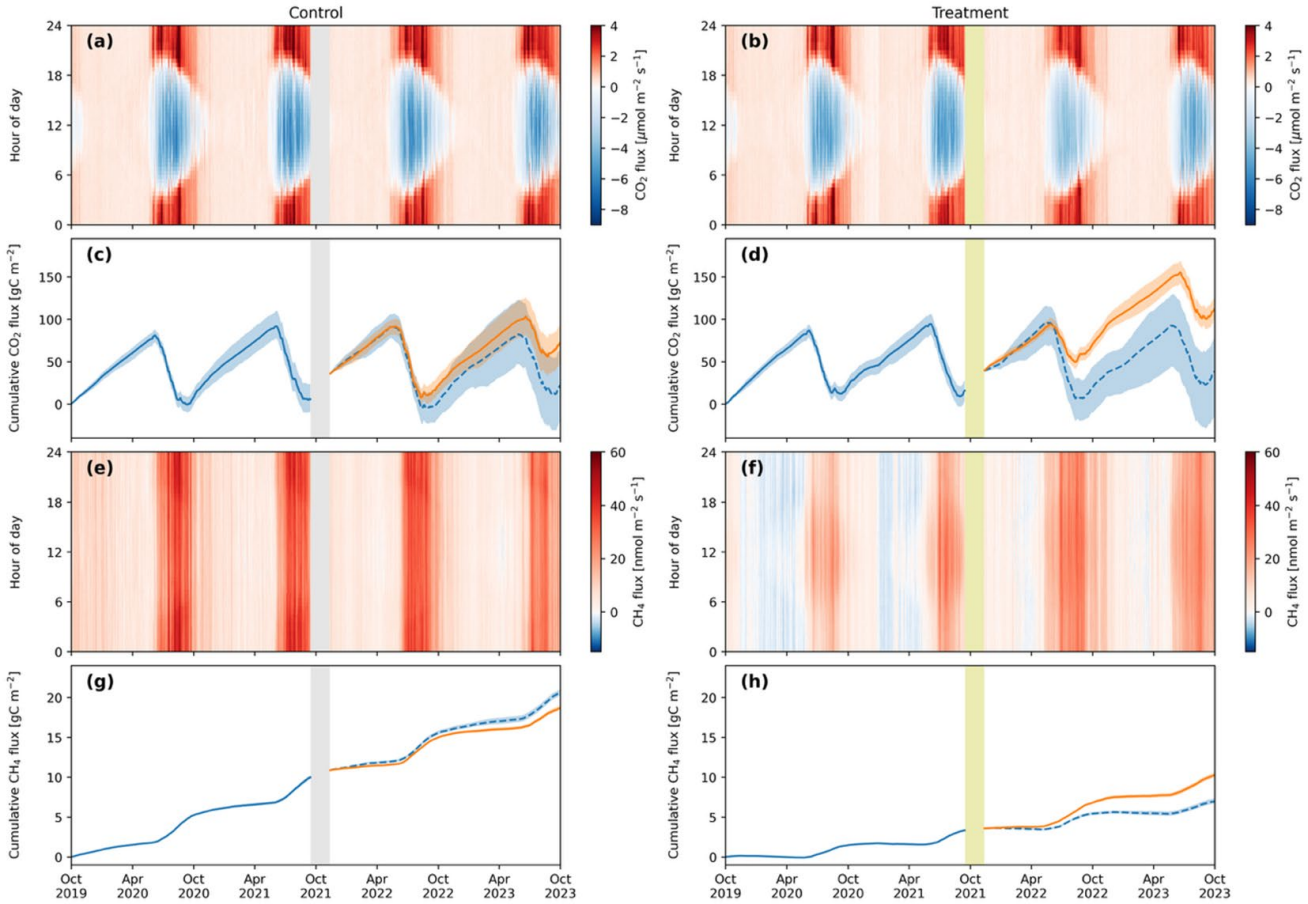
CO_2_ fluxes at the Control Site (**a**) and Treatment Site (**b**) in 30 min intervals by hour of the day; cumulative CO_2_ fluxes at the Control Site (**c**) and Treatment Site (**d**); CH_4_ fluxes at the Control Site (**e**) and Treatment Site (**f**) in 30 min intervals by hour of the day; and cumulative CH_4_ fluxes at the Control Site (**g**) and Treatment Site (**h**).In c, d, g, and h the solid blue lines are the Pre-Treatment Models, the orange lines are the Treatment Models, and the dashed blue lines are the Pre-Treatment Model simulations of Treatment Period fluxes (i.e., a predicted flux for the Control Site (**c**,** g**) and a counterfactual flux for the Treatment Site (**d**,** h**)). In c, d, g, and h the shaded areas indicate the 90th percentile range from the 5th percentile to 95th percentile. The yellow bar represents the rewetting time period at the Treatment Site in September to November of 2021. The grey bar on the Control Site plots indicate the same time period and is included to indicate that the flux data at the Control Site was processed in exactly the same manner as the Treatment Site. The Control Site remains drained and was unaffected by the rewetting of the Treatment Site.

Over the four years of the study at the Control Site the growing season CO_2_ flux ranged from − 7.5 µmol m^− 2^s^− 1^ during the daylight hours to 5.0 µmol m^− 2^s^− 1^ at night (Fig. 5a; Table 3). Before the rewetting at Treatment Site the growing season CO_2_ flux ranged from − 6.3 µmol m^− 2^s^− 1^ during the daylight hours to 5.9 µmol m^− 2^s^− 1^ at night. After the rewetting at Treatment Site the growing season CO_2_ flux ranged from − 5.9 µmol m^− 2^s^− 1^ during the day to 5.5 µmol m^− 2^s^− 1^ at night.

In the two years before the rewetting, the mean growing season CO_2_ flux during daylight hours was − 2.2 µmol m^− 2^ s^− 1^ at the Control Site and − 2.0 µmol m^− 2^ s^− 1^ at the Treatment Site (Fig. 5b). In the first year after the rewetting, the mean growing season CO_2_ flux during the daylight hours was − 1.8 µmol m^− 2^ s^− 1^ at the Control Site and − 1.1 µmol m^− 2^ s^− 1^ at the Treatment Site. In the second year after the rewetting, the growing season mean CO_2_ flux during the daylight hours was − 1.89 µmol m^− 2^ s^− 1^ at the Control Site and − 1.90 µmol m^− 2^ s^− 1^ at the Treatment Site. During the non-growing season, the mean CO_2_ flux at all hours of the day were similar at both Treatment Site and Control Site across all four years of the study, ranging from from 0.29 to 0.33 µmol m^− 2^s^− 1^.

#### Cumulative CO_2_ fluxes

The cumulative CO_2_ flux after one year at the Control Site was 8.2 **±** 9 g C m^− 2^, 11.8 **±** 17 g C m^− 2^ after two years, 18.8 **±** 11 g C m^− 2^ after three years, and 69.0 **±** 24 g C m^− 2^ after four years (Fig. 5c). The Pre-Treatment Model predicting Treatment Period fluxes estimated that after four years the cumulative flux would be 22.4 **±** 49 g C m^− 2^, lower than but within the uncertainty range of the best estimate of the actual cumulative flux (69.0 **±** 24 g C m^− 2^) at this time.

The cumulative CO_2_ flux after one year at the Treatment Site was 21.5 ± 9 g C m^− 2^, 24.3 ± 13 g C m^− 2^ after two years, 77.6 ± 9 g C m^− 2^ after three years, and 118.8 ± 15 g C m^− 2^ after four years (Fig. 5d). The Pre-Treatment Model predicting Treatment Period fluxes estimated that had the rewetting not occurred the cumulative flux would have been 38.7 ± 47 g C m^− 2^ after four years. Therefore, the counterfactual modeling estimated that the rewetting increased the CO_2_ flux by 80.3 ± 49 g C m^− 2^ in the two years after the restoration (Table 5).

#### Annual CO_2_ fluxes

The mean annual CO_2_ flux at the Control Site across the four years of the study was 17.3 ± 10 g C m^− 2^ yr^− 1^ and ranged from 3.6 ± 20 g C m^− 2^ yr^− 1^ (second year) to 50.2 ± 26 g C m^− 2^ yr^− 1^ (fourth year) (Table 4). At the Treatment Site the mean annual CO_2_ flux was 12.2 ± 3.8 g C m^− 2^ yr^− 1^ before the rewetting and 47.3 ± 5.4 g C m^− 2^ yr^− 1^ after the rewetting. The highest annual CO_2_ flux at the Treatment Site was 53.3 ± 13 g C m^− 2^ yr^− 1^ in the year after the rewetting. In the following year, the fourth year of the study, the annual CO_2_ flux decreased to 41.2 ± 18 g C m^− 2^ yr^− 1^.

### Vertical CH_4_ fluxes

#### Instantaneous CH_4_ fluxes

Across all four years of the study, CH_4_ fluxes at the Control Site ranged from − 2.8 nmol m^− 2^ s^− 1^ to 50.7 nmol m^− 2^ s^− 1^ (Fig. 5e). Before the rewetting the CH_4_ fluxes at the Treatment Site ranged from − 5.9 nmol m^− 2^ s^− 1^ to 35.9 nmol m^− 2^ s^− 1^, and after the rewetting CH_4_ fluxes ranged from − 3.4 nmol m^− 2^ s^− 1^ to 35.5 nmol m^− 2^ s^− 1^ (Fig. 5f).

Across all years of the study, the mean CH_4_ flux at the Control Site during the growing season was 28.0 nmol m^− 2^ s^− 1^ and during the non-growing season was 7.2 nmol m^− 2^ s^− 1^ (Fig. 5e). Before the rewetting at the Treatment Site the mean CH_4_ flux was 14.6 nmol m^− 2^ s^− 1^ during the growing season and 0.5 nmol m^− 2^ s^− 1^ during the non-growing season (Fig. 5f). After the rewetting at the Treatment Site the mean CH_4_ flux was 20.0 nmol m^− 2^ s^− 1^ during the growing season and 6.25 nmol m^− 2^ s^− 1^ during the non-growing season.

#### Cumulative CH_4_ fluxes

The cumulative CH_4_ flux after one year at the Control Site was 5.2 **±** 0.11 g C m^− 2^, 10.3 **±** 0.25 g C m^− 2^ after two years, 14.9 **±** 0.14 g C m^− 2^ after three years, and 18.5 ± 0.31 g C m^− 2^ after four years (Fig. 5g). The Pre-Treatment Model predicting Treatment Period fluxes estimated that after four years the cumulative flux would be 20.6 ± 0.43 g C m^− 2^, slightly higher than the estimate of the actual flux at this time.

The cumulative CH_4_ flux after one year at the Treatment Site was 1.5 ± 0.06 g C m^− 2^ yr^− 1^, 3.5 ± 0.14 g C m^− 2^ after two years, 7.3 ± 0.25 g C m^− 2^ after three years, and 10.7 ± 0.37 g C m^− 2^ after four years (Fig. 5h). The Pre-Treatment Model predicting Treatment Period fluxes estimated that had the rewetting not occurred the cumulative flux would have been 7.0 ± 0.03 g C m^− 2^, lower than the actual flux at this time. Therefore, the counterfactual modeling estimated that the rewetting increased the CH_4_ flux by 3.7 ± 0.37 g C m^− 2^ in the two years after the restoration (Table 5).

#### Annual CH_4_ fluxes

The mean annual CH_4_ flux at the Control Site across the four years of the study was 4.6 ± 0.1 g C m^− 2^ yr^− 1^ and ranged from 5.2 ± 0.1 g C m^− 2^ yr^− 1^ (first year) to 3.6 ± 0.3 g C m^− 2^ yr^− 1^ (fourth year) (Table 4). At the Treatment Site the mean annual CH_4_ flux was 1.8 ± 0.04 g C m^− 2^ yr^− 1^ before the rewetting and 3.6 ± 0.13 g C m^− 2^ yr^− 1^ after the rewetting. The highest annual CH_4_ flux at the Treatment Site was 3.8 ± 0.3 g C m^− 2^ yr^− 1^ in the year after the rewetting. In the following year, the CH_4_ flux was 3.4 ± 0.4 g C m^− 2^ yr^− 1^.

**Table 3:** The growing season is defined as starting on the date at which each peatland site transitioned from being a CO_2_ source to a CO_2_ sink in the early summer and ending on the date at which each peatland site transitioned from being a CO_2_ sink to CO_2_ source.

Year	Growing season	
	Treatment	Control
2020	7 June − 13 September	6 June − 20 September
2021	6 June − 5 September	7 June − 9 September
2022	1 May − 11 August	8 May − 11 August
2023	22 June − 25 August	19 June − 24 August

**Table 4:** Annual CO_2_ and CH_4_ fluxes at the Control Site and the Treatment Site in g C m^− 2^ using the Pre-Treatment Models for the Pre-Treatment Period and the Treatment Models for the Treatment Period. The table shows the best estimate of the annual fluxes at both sites over the four years of the study.

Year	Control Site		Treatment Site	
	CO_2_-C	CH_4_-C	CO_2_-C	CH_4_-C
	g C m^− 2^ yr^− 1^		g C m^− 2^ yr^− 1^	
2019/20	8.2 ± 9	5.2 ± 0.1	21.5 ± 9	1.5 ± 0.1
2020/21	3.6 ± 20	5.1 ± 0.3	2.8 ± 12	2.0 ± 0.2
2021/22	7.0 ± 21	4.6 ± 0.3	53.3 ± 13	3.8 ± 0.3
2022/23	50.2 ± 26	3.6 ± 0.3	41.2 ± 18	3.4 ± 0.4
Mean	17.3 ± 10	4.6 ± 0.1	Before^1^: 12.2 ± 3.8 After^2^: 47.3 ± 5.4	Before^1^: 1.8 ± 0.04 After^2^: 3.6 ± 0.13

^1^2-year mean before the rewetting.

^2^2-year mean after the rewetting.

**Table 5:** The effect of the rewetting as estimated by the BART counterfactual modeling one and two years after the rewetting. The effect is estimated by taking the difference between the cumulative fluxes of the Treatment Model and the Pre-Treatment Model simulations at the Treatment Site during the Treatment Period on 1 October 2022 (one year after the rewetting) and 1 October 2023 (two years after the rewetting). Positive values imply the rewetting is estimated to have caused emissions of CO_2_ and CH_4_ to increase.

	Treatment Site (estimated effect of rewetting)	
	CO_2_-C	CH_4_-C
	g C m^− 2^	
One year after rewetting	57.6 ± 29	1.9 ± 0.30
Two years after rewetting	80.3 ± 49	3.7 ± 0.47

### Lateral fluxes

#### DOC concentrations

In 2021, the mean DOC_C_ across the four sampling dates was 28.8 mg l^− 1^ at the Control Site and across the three sampling dates in 2022 was 23.3 mg l^− 1^ at the Treatment Site (Table S2). In 2022, the mean DOC_C_ across the three sampling dates was 34.0 mg l^− 1^ at the Control Site and 41.7 mg l^− 1^ at the Treatment Site (Table S2).

#### Lateral DOC carbon flux estimates

The estimated discharge from the Control Site was 35 900 m^3^ in 2021 and 26 500 m^3^ in 2022 (Table S3). The estimated lateral DOC_f_ from the Control Site was 17 g C m^− 2^ yr^− 1^ in 2021 and 19 g C m^− 2^ yr^− 1^ in 2022.

The estimate discharge from the Treatment Site was 54 800 m^3^ in 2021 and 40 500 m^3^ in 2022 (Table S3). The estimated lateral DOC_f_ from the Treatment Site was 14 g C m^− 2^ yr^− 1^ in 2021 and 15 g C m^− 2^ yr^− 1^ in 2022.

## Discussion

### Distinguishing between short-term and long-term effects of rewetting

This paper reports four years of flux data from 2019 to 2023, including two years of the flux data from the Treatment Site prior to the rewetting, two years of flux data from the Treatment Site after the rewetting, and four years of flux data from the Control Site during which the site remained in the drained condition. With only two years of flux data after rewetting at the Treatment Site, our results reflect only the short-term effects of rewetting. Monitoring at both sites is ongoing to collect a time series of flux data that will allow for the assessment of the longer-term effects of rewetting.

### Vertical and lateral carbon flux dynamics

In reports of the land sector carbon budget to the United Nations Framework Convention on Climate Change (UNFCCC), Norway uses IPCC Tier 1 coefficients to estimate greenhouse gas emissions from drained peatlands. The Tier 1 CO_2_ emission coefficient for a drained nutrient poor inland organic soil that is forest, shrubland, and non-forest in a drained condition is 37 g C m^− 2^ yr^− 1^^22^. By comparison the mean CO_2_ flux at the Control Site across the four years of the study was 17.3 ± 10 g C m^− 2^ yr^− 1^, a slightly lower-than-expected mean value. However, the variability in the annual flux of CO_2_ from the Control Site was higher than expected. In the first three years of the study the Control Site was nearly CO_2_ neutral. However, in the fourth year of the study the CO_2_ fluxes increased to 50.2 ± 26 g C m^− 2^ yr^− 1^. To our knowledge, the rewetting of the Treatment Site did not have an effect on the Control Site, making this increase unexpected. The annual mean water table depth at the Control Site remained unchanged throughout the study and annual rainfall totals in the first three and last year of the study were similar. The reason for the observed increase in CO_2_ fluxes from the Control Site in the last year of the study is unclear, and a longer time series of flux data is needed to explain this increase.

The Treatment Site in the two years before the rewetting also had a lower-than-expected mean CO_2_ flux of 12.2 ± 3.8 g C m^− 2^ yr^− 1^. In the first year after the rewetting the CO_2_ flux at the Treatment Site increased to 53.3 ± 13 g C m^− 2^ yr^− 1^, which was likely related to the soil and vegetation disturbances caused by the rewetting activities. During the rewetting, trees growing in the ditches were cut down while the low-lying vegetation was mechanically removed from the ditches. Peat was then excavated from the areas between the ditches to fill the ditches, and then the vegetation was replaced. While the flux data was not formally partitioned in this study, the felling of trees and movement and replacement of low-lying vegetation appear to have reduced plant CO_2_ uptake. In addition, the soil excavations likely lead directly to an increase in the labile carbon pool and a higher rate of organic matter mineralization. In the second year after the rewetting the annual CO_2_ flux decreased to 41.2 ± 18 g C m^− 2^ yr^− 1^. Ongoing monitoring at the site will determine if and by how much CO_2_ emissions at the Treatment Site continue to decrease in future years.

Short-term increases in CO_2_ and CH_4_ fluxes after the completion of rewetting projects, like those observed at the Treatment Site in this study, are well documented^50^. However, it is the longer-term effects of rewetting on carbon fluxes that are most important when considering the carbon balance of such rewetting projects. A growing number of studies indicate that the majority of restored temperate and boreal peatlands return to CO_2_ sinks on time scales of approximately twenty years^50–52^. However, in some cases, rewetting has not returned previously drained peatland sites to a CO_2_ sink within this time period^52^. Additionally, Wilson et al.^52^ state that CO_2_ fluxes from restored peatlands may be more sensitive to inter-annual variability in weather conditions. Thus, previous research suggests CO_2_ fluxes at the Treatment Site are likely to continue to gradually decrease, but that the annual flux variability may be high, especially in comparison to pristine peatlands.

Overall, the CO_2_ fluxes from both sites in this study, including the Treatment Site after rewetting, were relatively weak CO_2_ sources. Ojanen et al.^53^ estimated CO_2_ fluxes at 68 drained for forestry peatland sites in Finland by subtracting litter input to soil from CO_2_ soil efflux and found that nutrient rich sites were on average CO_2_ sources of 142 g C m^− 2^ yr^− 1^ and nutrient poor sites were on average CO_2_ sinks of -52 g C m^− 2^ yr^− 1^. As such, the range of CO_2_ fluxes of both sites in this study across all years, which ranged from 3.6 ± 20 to 53.3 ± 13 g C m^− 2^ yr^− 1^, were higher than CO_2_ fluxes found on nutrient poor forestry-drained peatlands in Finland, but was well within the range of what has been found in Finland on forestry-drained peatlands overall.

The mean annual CH_4_ flux at the Control Site was 4.6 ± 0.1 g C m^− 2^ yr^− 1^, while the mean annual CH_4_ flux at the Treatment Site was 1.8 ± 0.04 g C m^− 2^ yr^− 1^ before the rewetting. Thus, the CH_4_ flux at the Control Site was more than double that of the Treatment Site during the Pre-Treatment Period. The water table level of both sites before the rewetting was similar and the vegetation type of the two sites were also similar. This study did not analyze carbon fractions, but it is possible that the Control Site had a higher percentage of labile carbon than the Treatment Site. Before the rewetting there was also a weak uptake of CH_4_ at the Treatment Site in the non-growing season. Methane oxidation that results in CH_4_ uptake in drained peatlands has also been observed in several other studies^54,55^. The strength of this uptake in the non-growing season weakened with the rewetting, but surprisingly was still occurring. Future work at the site would also benefit from soil analyses that measured the nutrient content of the peat at both sites. Incubation experiments could also be performed on peat from the site to study near surface CH_4_ oxidation, which can be an important determinant of overall CH_4_ fluxes^56^.

Annual CH_4_ fluxes increased after the rewetting at the Treatment Site to a two-year mean of 3.6 ± 0.13 g C m^− 2^ yr^− 1^, which was double the mean flux before rewetting. The increase in CH_4_ emissions will likely decrease through time as observed in Kalhori et al.^51^. In addition, modeling work indicates that short-term increases in CH_4_ emissions from rewetted peatlands do not offset the benefits of decreased CO_2_ emissions when multi-decadal or century long time scales are considered^5,57^.

Our rough estimates of DOC export, however crude given the low number of samples collected and the greatly simplified methodology used for estimating discharge, suggest that lateral carbon transport from the peatland sites is possibly a significant contributor to the net ecosystem carbon balance of both sites. DOC is commonly significant to the net ecosystem carbon balance in peatland ecosystems, and especially of drained peatlands^20,58^. The DOC export estimates in this study ranged from 14 to 19 g C m^− 2^ yr ^− 1^, which are the same order of magnitude as the vertical carbon fluxes observed at both sites. The IPCC Tier 1 estimate of DOC export from drained boreal peatlands is 13 g C m^− 2^ yr ^− 1^, while the estimate for natural boreal peatlands is 8 g C m^− 2^ yr ^− 1^^22^. Given these IPCC estimates, it seems likely that the Control Site DOC export will remain similar in future years, while the Treatment Site DOC flux may start to slowly decline as the site recovers from the disturbance caused by the rewetting. Haapalehto et al.^59^ found that after rewetting of boreal peatlands in Finland, DOC levels take at least 5 years to decrease from pre-rewetting levels, while peatland hydrology takes roughly 10 years to approximate pristine peatland hydrology.

### Water table depth and implications for carbon fluxes

The median water table depth of both the Control Site and of the Treatment Site before rewetting was approximately 30 cm, and after the rewetting the median water table depth at the Treatment Site rose to 20 cm. At three forestry-drained peatland sites in central and southeastern Norway, Stachowicz et al.^60^ measured water table depth both before and after rewetting. The authors found that before rewetting the water table depths were 20, 23, and 30 cm, and thus overall, slightly shallower than the water table depths at the Control Site and Treatment Site in this study. After rewetting of the three sites, which was also achieved by ditch blocking, Stachowicz et al.^60^ found that the water table depth rose by an average of 6 cm, and 8 cm at the study site most similar two sites in this study. This result is very similar to the change in water table depth after rewetting at the Treatment Site in this study of 10 cm. As such, it appears that the drainage conditions of the two sites in this study, and the effects of the rewetting on water table depth at the Treatment Site, are similar to other sites recently rewetted in southern and central Norway.

To upscale carbon fluxes from drained peatlands, it is increasingly common to use water table depth transfer functions. Koch et al.^61^ developed such relationships for drained peat soils in Denmark, Evans^62^ did so for the UK, and Tiemeyer et al.^63^ for Germany. However, applying these transfer functions to the Control Site in this study gives a mean annual CO_2_ flux ranging from of 100 to 900 g C m^− 2^ yr^− 1^, and thus greatly overestimates the actual mean annual CO_2_ fluxes at the Control Site in this study of 17.3 ± 10 g C m^− 2^ yr^− 1^. This overestimation is likely because these relationships were not developed specifically for forestry-drained peat soils. Conversely, Ojanen and Minkkinen^64^ developed a water table depth to carbon flux transfer function for forestry-drained boreal peatlands in Finland. This transfer function estimates an annual flux of -17 g C m^− 2^ yr^− 1^, and thus much more closely approximates the observed fluxes at the site (underestimates by 34 g C m^− 2^ yr^− 1^). Developing similar transfer functions for peatlands drained for forestry in Norway is currently not possible because of a lack of carbon flux data. To our knowledge, this is the first study to report CO_2_ and CH_4_ fluxes from a forestry-drained peatland in Norway.

### Vegetation dynamics

The vegetation mapping was conducted after the rewetting of the Treatment Site, but the mapping of the Control Site revealed that only 20% of the Control Site flux tower footprint was classified as drained fen vegetation, while 69% of the footprint was fen vegetation associated with nutrient poor pristine fens. Vegetation mapping using the *Nature in Norway* system is a binary typology in which the peatland is mapped as either a drained fen vegetation type or as a pristine fen vegetation type. The mapping system does not have vegetation types for transitional states between pristine and drained fen vegetation. The presence of *Betula* (birch) and *Pinus* (pine), together with the coverage of shrubs, and less dominance of *Sphagnum* mosses, were used for mapping of drained fen vegetation. The sites in this study appear to have been drained effectively enough to support greater tree cover than they would have under undrained conditions but not drained effectively enough for the afforestation efforts to be successful. Nutrient poor peatlands drained for forestry in Finland have been found to typically be carbon sinks^53^, however in this study the Control Site and the Treatment Site before rewetting were both weak carbon sources. This phenomenon may be explained by the fact that the sites in this study were very slowly afforesting and thus the tree growth was not fast enough to turn the sites into carbon sinks as has been observed in Finland.

The exact date that the two sites were drained is unknown due to a lack of historical records and can only be constrained to sometime between 1956 and 1975 using ariel photography. Despite it being at least 50 years since the sites had been drained, the canopies of both sites remained open at the start of this project in 2019, with only discontinuous coverage of birch (*Betula pubescens*) and pine (*Pinus sylvestris*). Forestry drained peatlands in the Nordic countries typically have a 60–100 stand rotation time^64^ during which the ditches are commonly cleaned multiple times^65^. It is not clear when the afforestation efforts at the Treatment Site and Control Site were abandoned. Whether the sites were sowed or planted after ditching, or if the ditches were ever maintained is unknown.

### The BART model

To the best of our knowledge, this study is the first time that Bayesian Additive Regression Tree (BART) models have been applied to eddy covariance data. The BART models used in this study were adopted because they warrant more testing in geoscientific applications, provide comparatively easily attainable uncertainty estimates, and can be used to estimate causal effects.

Note that, other than the hyperparameters of the Particle Gibbs sampler (number of chains, steps, and burn-in), the BART models employed here only have two hyperparameters: m (the number of regression trees) and the prior scale of the standard deviation. Thus, like other decision tree-based methods, they require minimal input from the user while maintaining a high predictive performance. At the same time, the fact that BARTs are Bayesian models make them less prone to overfit and provide valuable uncertainty quantification^41^. Although more flexible probabilistic machine learning models such as Bayesian neural networks^39^ exist for flux estimation, their added flexibility comes at the cost of being considerably more challenging to set up than BARTs.

Of the nine environmental variables that the BART models in this study used to predict CO_2_ and CH_4_ fluxes, water table depth was not one of them despite water table depth being one of the most important environmental variables used in the peatland carbon flux modeling^66^. Water table depth was omitted so that the Pre-Treatment Model could predict the counterfactual Treatment Period fluxes at the Treatment Site. Including actual water table depth, which significantly changed during the Treatment Period, would interfere with the estimation of counterfactual fluxes. In future work, hydrological modeling could estimate the water table depth of the Treatment Site had it not been restored and these estimations could be used in the counterfactual BART modeling, which might improve the Pre-Treatment Models ability to predict Treatment Period fluxes. Because water table depth was omitted as a BART predictor the fluxes that are estimated by the Pre-Treatment Models in the Treatment Period are likely less accurate than they would have been had we modeled the counterfactual water table depth and included it as a predictor. We hypothesize that the Pre-Treatment Model would have more accurately predicted Treatment Period fluxes at the Control Site if water table depth had been included, which would have in turn increased our confidence in the accuracy of the counterfactual estimates at the Treatment Site.

Another limitation of our counterfactual modeling is that some of the other environmental variables used to force BART model simulations of the counterfactual fluxes may have been affected by the restoration, such as soil temperatures at five depths (5–70 cm) and vapor pressure deficit. However, the other environmental variables used to force the BART models including shortwave and longwave incoming radiation, air temperature and pressure, wind speed, and growing degree days, we expect to be minimally affected by the restoration or not affected at all (i.e., time since rain).

### Limitations and future work

The primary limitation of most peatland rewetting studies, including this study to date, is that the sites are only monitored for three or fewer years after rewetting, which is not sufficient to determine the steady state effect of the rewetting on the ecosystems carbon balance^17,51^. Monitoring at the sites is continuing so that a longer time series of CO_2_ and CH_4_ fluxes after the rewetting is recorded. An additional limitation of this study is that, while it included a drained peatland site as a control, it did not include a pristine reference peatland site. In future studies, monitoring a third, undisturbed peatland would strengthen the experimental design.

The great variation in vegetation composition, nutrient status, micrometeorology, hydrology, and land use history of peatlands make it difficult to predict carbon emissions of a specific drained peatland and how the specific peatland site will respond to rewetting^17^. In addition, Norwegian peatland ecosystems vary greatly by climate, proximity to the coast, and elevation^13^, and many of them are still not present on official maps^6^. However, predicting carbon emissions has been shown to be possible in other studies. By taking chamber-based CO_2_, CH_4_, and N_2_O efflux measurements at drained peatlands across Finland, Ojanen et al.^53^ were able to build a model that explained 66% of the variation in the total annual CO_2_ effluxes at 68 study sites across Finland. Conducting similar multi-site studies across drained Norwegian peatlands would help characterize carbon flux variability and could further assist in refining emissions estimates at the country level.

Overall, the DOC export estimates in this study were limited by a coarse estimation of discharge, sparse DOC measurements, and by solely considering DOC as the lateral carbon transport mechanism. We recommend that further work include the installation of v-notch weirs to track discharge from both the Treatment and Control Sites. In addition, hydrological models could be calibrated to these weir data so that discharge can be simulated in both past and future. A total of seven DOC measurements were made in this study, and these measurements did not span the entire snow free season. DOC measurements throughout the snow free season need to be taken to resolve the possible seasonal patterns in DOC concentration. Lastly, while DOC is often the most significant contributor to lateral carbon fluxes in drained organic soils^22^, dissolved inorganic carbon (DIC) and particulate organic carbon (POC), are also contributors to lateral carbon transport and also warrant being measured at both sites.

## Conclusion

Differences in CO_2_ and especially CH_4_ fluxes between the Control Site and Treatment Site before the rewetting meant that the Control Site could not serve as the counterfactual representation of the fluxes at the Treatment Site, as was originally intended by the experiment. However, the ability of the BART models to predict counterfactual carbon fluxes appears to be a promising alternative in determining the effect of rewetting on the net ecosystem carbon balance. Even though the Control Site did not serve directly as a counterfactual representation of the Treatment Site, tracking carbon fluxes at the Control Site is beneficial as it is currently the only drained for forestry peatland being actively monitored for carbon fluxes in Norway.

At both the Control Site and the Treatment Site, the mean annual CO_2_ fluxes were lower than - and CH_4_ fluxes were higher than - the IPCC Tier 1 emission coefficients for boreal drained nutrient poor organic soils. Export of DOC was similar in magnitude to the vertical CO_2_ and CH_4_ flux at both the Treatment Site and Control Site, warranting more accurate DOC estimates in the future.

The CO_2_ flux from the Treatment Site increased significantly after the rewetting but decreased from the first to the second year after rewetting. Previous peatland rewetting studies suggest that the carbon mitigation potential of peatland rewetting can take approximately 10–20 years to be realized^51^. However, in some cases, rewetting does not return peatland ecosystems to carbon sinks within this time period^67^. As such, monitoring at the Control Site and Treatment Site will continue to elucidate the carbon flux trajectory of both sites in the years to come.

## Supplementary Information

Below is the link to the electronic supplementary material.

Supplementary Material 1

## Data Availability

The BART gap-filled flux data, the nine environmental variables that were used as the BART predictors, and the water table depth data are all available for download at the following DOI: https://doi.org/10.5281/zenodo.17552123.
