# Supplementary material for: Carbon dynamics of a controlled peatland rewetting experiment in the Norwegian boreal zone
Source: Sci Rep. 2025 Dec 17;16:1216. doi: 10.1038/s41598-025-30836-2 (PMC12789543; doi:10.1038/s41598-025-30836-2)
Supplement: Supplementary file 1 — Supplementary Material 1 [file 41598_2025_30836_MOESM1_ESM.docx]

**Supplementary Information**

Section 1. Location of the water table depth monitoring wells

Five water table depth monitoring wells were installed at each site. Wells T1, T2, T3, T4, and T5 are at the Treatment Site and wells C1, C2, C3, C4, and C5 are at the Control Site.

Table S1. Location of the water table depth monitoring wells (WGS84) and distance of the monitoring wells to the center of the nearest drainage ditch (m).

| Well number | Longitude | Latitude | Distance to center of nearest drainage ditch (m) |
| --- | --- | --- | --- |
| T1 | 12.25445906 | 61.10514513 | 7.9 |
| T2 | 12.25466085 | 61.10522046 | 4.8 |
| T3 | 12.25459116 | 61.10525142 | 2.4 |
| T4 | 12.2548897 | 61.10516384 | 3.8 |
| T5 | 12.25513421 | 61.10512078 | 6.8 |
|  |  |  |  |
| C1 | 12.2508132 | 61.11180164 | 3.2 |
| C2 | 12.25088955 | 61.11168584 | 10.4 |
| C3 | 12.25087345 | 61.11155634 | 2.2 |
| C4 | 12.25060931 | 61.11153759 | 10.1 |
| C5 | 12.2503442 | 61.11157437 | 11.2 |

Section 2. BART model setup

The BART models represent observed fluxes as a data-generating model consisting of a sum of m = 300 regression trees, each dependent on the environmental inputs and internal parameters, and an observation error term. Instead of being optimized, the parameters of the regression trees and observation error are treated as random (i.e., uncertain) variables to be inferred through Bayesian inference. This was accomplished by specifying a prior distribution on these parameters. Following Chipman et al. [1], the regression tree parameters were assigned default BART priors. The observation errors were assumed to follow a zero-mean Gaussian distribution with an uncertain noise standard deviation (σ) to which a half-normal prior with a scale of 0.005 was assigned. This effectively defines a Gaussian likelihood, which, together with the prior over the tree and noise parameters, enables sampling from the posterior distribution using Bayesian inference.

Posterior sampling was performed using a Particle Gibbs sampler proposed by Lakshminarayanan et al. [2], which is a Particle Markov Chain Monte Carlo algorithm tailored for BARTs as implemented in PyMC [3, 4]. For sampling 20 parallel Markov chains were run each with 2 000 steps. For each chain, the first 1 000 steps are treated as a burn-in period and subsequently discarded to ensure a better approximation of the posterior distribution [3]. This leaves 20 000 sets of samples of tree and noise parameters from the posterior distribution. One in every 100 of these parameter sets is selected such that 200 parameter sets compose the posterior ensemble, which together define our trained BART. Based on this trained BART model, posterior flux predictions can be made for the entire measurement period, including periods with gaps in the flux data as well for counterfactual flux estimations.

Section 3. Estimate of DOC concentrations

Water samples were collected by hand in 0.5 L bottles on seven different occasions. Four sampling trips were made before the rewetting in 2021 (June 22, and July 26, 28, 30), and three after the rewetting in 2022 (September 9, 13, and October 10).

Table S2. DOC concentrations (DOC_c_) at the control and treatment sites in 2021 and 2022.

|  | DOC_c_ (mg l^-1^) | |
| --- | --- | --- |
| Date | Control | Treatment |
| 22 June 2021 | 30.8 | 29.8 |
| 26 July 2021 | 23.0 | 13.8 |
| 28 July 2021 | 22.6 | 14.6 |
| 30 July 2021 | 38.8 | 34.8 |
| **2021 mean** | **28.8** | **23.3** |
| 9 Sept. 2022 | 29.8 | 43.0 |
| 13 Sept. 2022 | 41.0 | 48.0 |
| 10 Oct. 2022 | 31.0 | 34.0 |
| **2022 mean** | **34.0** | **41.7** |

Section 4. Estimate of discharge volume

Discharge volume of both peatland sites was estimated using a simple water balance equation (Eq. 1).

(Eq.1) $Q=\frac{\left( P-ET \right)}{1000}*A$

Where Q is discharge (m^3^ yr^-1^), P is precipitation (mm yr^-1^), ET is evapotranspiration (mm yr^-1^), and A is the area covered by the drainage ditches including a 10 m buffer (m^2^). ET was estimated with the flux tower at the Control Site and precipitation data was taken from the nearest weather station, SN180 Trysil vegstasjon [5]. P and ET were assumed to be the same at the Treatment Site and Control Site. Catchment area was determined by analyzing a DEM [6] in ArcMap 10.8 [7].

Table S3. Annual precipitation, evapotranspiration, and discharge estimates from 2019 to 2022.

| Year | Precipitation (mm) | Evapotranspiration (mm) | Discharge (mm) | Treatment site discharge (m^3^) | Control site discharge (m^3^) |
| --- | --- | --- | --- | --- | --- |
| 2021 | 984 | 192 | 792 | 54 800 | 35 900 |
| 2022 | 782 | 197 | 585 | 40 500 | 26 500 |

**References**

1. Chipman, H.A., George, E.I., McCulloch, R.E. (2010). BART: Bayesian Additive Regression Trees. *The Annals of Applied Statistics*. Vol. 4. No. 1. 266-298. doi:10.1214/09-AOAS285
2. Lakshminarayanan, B., Roy, D. & Teh, Y.W. Particle Gibbs for Bayesian Additive Regression Trees. Proceedings of the Eighteenth International Conference on Artificial Intelligence and Statistics, PMLR 38:553-561, 2015. (2015).
3. Martin O.A., Ravin, K., Junpeng, L. (2021). Bayesian Modeling and Computation in Python Boca Ratón. ISBN 978-0-367-89436-8
4. Quiroga, M., Garay, P.G., Alonso, J.M., Loyola, J.M., Martin, O.A. (2023). Bayesian additive regression trees for probabilistic programming, arXiv, doi:10.48550/arXiv.2206.03619
5. Norwegian Meteorological Institute (2023). Frost API. License: NLOD 2.0. Available at frost.met.no
6. Kartverket (2023). Digital elevation model of the Hisåsen site. Accessed via hoydedata.no. License: CC BY 4.0.
7. Environmental Systems Research Institute (ESRI). (2021). ArcGIS release 10.8. Redlands, CA. https://www.esri.com/en-us/arcgis/products/arcgis-desktop/overview
